# Ability of Emotional Regulation and Control as a Stress Predictor in Healthcare Professionals

**DOI:** 10.3390/ijerph20010541

**Published:** 2022-12-29

**Authors:** Marija Kadović, Štefica Mikšić, Robert Lovrić

**Affiliations:** 1 Faculty of Medicine, Josip Juraj Strossmayer University of Osijek, 31000 Osijek, Croatia; 2 Nursing Institute “Professor Radivoje Radić”, Faculty of Dental Medicine and Health Osijek, Josip Juraj Strossmayer University of Osijek, 31000 Osijek, Croatia

**Keywords:** emotional regulation, work-related stress, psychological support system, emotional stress, institutional obligations, healthcare professionals, quality of healthcare

## Abstract

Emotional Regulation and Control implies a person’s ability to respond to stressful demands and emotional experiences in a socially acceptable and adaptive way. The aim of this cross-sectional study was to examine the contribution of the ability of emotional regulation and control in the prediction of workplace stress in healthcare professionals. The study included 203 healthcare professionals employed at a hospital in the Republic of Croatia. Data were collected using two validated questionnaires: Questionnaire on Workplace Stressors for Hospital Professionals and Emotional Regulation and Control Questionnaire (ERC). Most respondents (64%) experienced stress in Workplace Organization and Financial Issues factor, while 52.7% experienced stress in Public Criticism factor. The respondents assessed their ability of emotional regulation and control to be low (mean = 55; range = 20–100). The level of experienced stress was significantly higher if the ability of emotional regulation and control was low (Spearman’s Rho = 0.308; *p* < 0.001). The multivariate regression model (11.2% explained variances; *p* = 0.001) indicated a greater possibility of severe stress in respondents who have stronger Memory of Emotionally Saturated Content (odds ratio = 1.18; 95% CI = 1.07–1.30). The results of this study signify the need to establish effective institutional support aimed at objectifying stress and strengthening emotional intelligence and empathy in healthcare professionals.

## 1. Introduction

### 1.1. Workplace Stress

Workplace stress is an issue often associated with employees’ quality of life in different professions [1]. Workplace stress is a specific type of stress whose source is in the work environment. The source of stressors can be the nature of the job itself, workplace Organization and working conditions [2]. Individual assessment of an objective state or event greatly influences a stress response in an individual [2]. An individual’s reaction to stress is the result of the interconnection of individual sensitivity, external circumstances, and stressors. Individual sensitivity is determined by personality, age, and lifestyle. External circumstances include environment, family, friends, and work environment [2]. One of the most famous theories of stress, the Lazarus stress theory [3], explains stress through two processes: cognitive appraisal by which an individual determines an event as threatening, and coping, which is the individual’s response to a perceived threat. One of the coping functions is controlling or changing the problem, which is more likely to be chosen if the person estimates that threatening environmental conditions can be changed. Otherwise, another coping function is more likely to be chosen, such as managing emotional reactions [1,3]. Workplace organization, career progression, individual role, work tasks, work environment, working conditions, and shift work belong to the most important group of work stressors [4]. The majority of them cannot be changed by an individual, but one should learn to manage emotional reactions well. Long-term exposure to stressors can lead to a disorder known as burnout syndrome. It is characterized by psychological, physical and/or psychophysical exhaustion [4]. Therefore, stressors related to the scope and unpredictability of work and the feeling of inadequate compensation are prevalent in the healthcare system. It is interesting that stressors related to mutual communication within the team are assessed relatively low [4]. Similar experiences were described in a study conducted among healthcare professionals, where it was found that the prevalence of workplace stress was as high as 68.2%, and that healthcare professionals working 50 or more hours a week or in night shifts are more prone to the negative consequences of stress and more inclined to evaluate different aspects of workplace as the sources of stress [4]. The hierarchy of stressor groups is the same in men and women, whereas women experience poor organization and insufficient finances, dangers and harms, and increased professional demands more stressful than men. On the other hand, exposure to public criticism and lawsuits and the impact of shift work are more stressful for men [2]. Additionally, there is a growing number of studies that confirm the fact that workplace stressors have an impact on the ability of emotional regulation and control in healthcare professionals [1,4,5,6].

### 1.2. Emotional Regulation and Control

Emotional regulation is defined as a set of processes by which a person tries to influence what emotions they will experience and express, at what time and in what way [5,7]. It enables a person to adjust the expression of their own emotions to the demands of the environment, as well as to protect, restrain and direct unpleasant emotions to avoid interference with personal functioning [8]. Although the concept of emotional regulation is interpreted in different ways, it can be said that it encompasses the ability to respond to stressful demands and emotional experiences in a socially acceptable, adaptive, and flexible way [8]. Emotional regulation includes the management of negative emotions and emotional reactions, the analysis of the reason causing emotion, the choice of reaction, as well as the ability to postpone immediate gratification. Therefore, it determines an individual’s external behavior and internal well-being [8]. In this context, the fact that clarity of feelings appears to be the opposite of emotional ambivalence stands out as particularly interesting [9]. Clarity of feelings was also a key concept in the experiment where negative moods were first induced and then the course of recovery was followed. Clarity of feelings was recognized as the prerequisite for effective mood management, i.e., people who understand their emotions more clearly are able to quickly find strategies to cope with stress, and thus mitigate the negative effects of a stressful event more quickly [9].

Scientific literature review shows that despite numerous studies on the stress of healthcare professionals [1,8,9], there is still an insufficient number of studies examining the connection between the ability of emotional regulation and control and the stressors in healthcare professionals in hospital environment. This deficit can affect global understanding of the importance of the concept of emotional regulation and control in healthcare professionals under the influence of certain stressors. This study aims to overcome this deficiency by providing results that will offer a deeper insight into the mechanism of emotional regulation and control abilities used by healthcare professionals and into workplace stressors in healthcare institutions.

Therefore, the main aim of this study was to examine the prevalence and level of work-related stress and the ability of emotional regulation and control in healthcare professionals in the mentioned hospital. In addition, the aim of this study was to examine to what extent the ability of emotional regulation and control contributes to explaining the prevalence of stress experienced by the respondents.

## 2. Materials and Methods

### 2.1. Study Design

This cross-sectional study was conducted in May 2022 at the Clinical Hospital Center in the Republic of Croatia at four departments where acute and/or chronic diseases patients are treated (ICU, Emergency Room, Psychiatry, Oncology). The study was conducted in one phase which included simultaneous examination of workplace stressors and emotional regulation and control in health professionals at the mentioned healthcare institution. The selection criteria for the healthcare institution included: (a) healthcare institutionis the largest and central healthcare institution in this region of Croatia, (b) healthcare institution belongs to the first category according to the Croatian classification (a national hospital with at least three clinics where the most complex diagnostic and therapeutic procedures are performed), (c) approval of the Hospital Board of Directors to conduct the study, (d) institution is a teaching base of the faculty within which the researchers conduct the study. The criterion for selecting the hospital departments for the study meant that 24 h service is provided. 

### 2.2. Respondents 

203 healthcare professionals (physicians, nurses, and midwives) were included in the study. The inclusion criteria were: (a) respondents are healthcare professionals permanently employed at the mentioned health institution, (b) respondents provide direct healthcare to hospitalized patients, (c) respondents read and understand the Croatian language, and (d) respondents voluntarily participate in the study. The sample size was calculated using the G*Power software (version 3.1.2, Franz Faul, University of Kiel, Germany). To observe a medium effect (q = 0.6) between two variables, with a significance level of 0.05 and a power of 0.80, the minimum required sample size was found to be 165 respondents. Sample size for the regression analysis with a power of 0.95 was found to be 164 respondents. Therefore, the minimum sample size was 180 respondents, which included additional 10% of respondents due to possible attrition. 

### 2.3. Instruments

The anonymous questionnaire used in this study included two standardized, validated instruments: (a) Questionnaire on Workplace Stressors for Hospital Professionals [10] and (b) Emotional Regulation and Control Questionnaire (ERC) [9]. 

The introductory part of the questionnaire contains questions about respondents’ sociodemographic characteristics (gender, age, level of education, occupation, professional degree, workplace, length of total employment, length of employment at the current workplace, working hours).

The structured Questionnaire on Workplace Stressors for Hospital Professionals by M. Milošević et al., written in Croatian [10], was based on the standardized Occupational Stress Questionnaire [11]. The fact that the original version of the questionnaire was psychometrically validated by the authors on the sample of 1481 healthcare workers (1086 nurses with various levels of education and 395 physicians) in four large hospitals in Croatia makes this questionnaire completely suitable choice regarding the context of this study and its respondents. Factor analysis of the original version of the questionnaire indicated six factors of high internal consistency type reliability (all Crombach α values were greater than 0.7). The instrument consists of 37 items/workplace stressors arranged into six domains (Table 1). The respondents assessed their experience of a certain stressor on the Likert scale: 1 (no stress), 2 (rarely stressful), 3 (sometimes stressful), 4 (stressful), 5 (highly stressful). The range of the total number of points is from 0–100 (calculation rule according to the author of the scale) where a higher number of points indicates a higher level of work-related stress in healthcare professionals. Following the guidelines of the authors of the original version of the questionnaire [9] during analyses (logistic regression) and interpretation of the results, the cut-off score was 60, when values ≤60 were interpreted as “No stress”, and values > 60 as “Stressful”. 

Reliability of factors and the overall questionnaire were tested using Cronbach’s coefficient. The reliability of each factor ranged from 0.796 (Professional and Intellectual Demands) to 0.892 (Public Criticism). The overall questionnaire reliability was 0.914, indicating high reliability.

Emotional Regulation and Control scale, designed by V. Takšić [9], is a part of the Emotional Regulation and Control Questionnaire (ERC). It was created by selecting items from the emotional regulation and control component, obtained by factoring self-assessment scales, and constructed for the purpose of operationalization and empirical tests of a model of emotional intelligence [9]. Furthermore, it contains statements aimed at assessing the (negative) effects of emotions and moods on thinking, memory, and behavior, as well as the ability to control emotions. Content of most scale statements refers to regulation and control of negative emotions and moods. The scale consists of a total of 20 items grouped into three domains/factors: (a) Effects of Emotions and Moods on Thinking and Behavior consisting of eight items, (b) Memories of Emotionally Saturated Content consisting of six items, (c) Control of Personal Emotional Reactions consisting of six items. Range of the total number of points on the ERC scale is from 20 to 100, where a higher number of points indicates a lower ability of emotional regulation and control in healthcare professionals.

Previous factor analyses of the ERIK scale show high correlations between factors. Authors Takšić et al. [12] tested different models of the factor structure of the original ERIK scale on nine different samples of respondents. The one-factor model mostly met the criteria and had satisfactory internal consistency with a reliability value of 0.816. However, the best agreement indices were shown by the three-factor structure model, with the problem of slightly weaker reliability values of the second (0.645) and third factor (0.583) [12]. Additionally, previous studies show that the reliability of the ERIK scale is significantly higher in samples of women than in samples of men, as well as in samples of university students than in samples of high school students [9].

Reliability of domains/factor and total ERC scale in this study was tested using Cronbach’s coefficient. Reliability was 0.696 in the domain Personal Emotional Reactions, 0.801 in the domain Memories of Emotionally Saturated Content, and 0.865 in the domain.

Effects of Emotions and Moods on Thinking and Behavior. The overall questionnaire reliability was 0.903, indicating high reliability.

### 2.4. Data Collection

Data was collected by submitting an online form (created using Alchemer survey software, Alchemer LLC comp., 168 Centennial Pkwy Ste 250 Louisville, CO, 80027-1257, USA) that included study details, guidelines, and questionnaire items. The respondents were sent an online form (link) via email. Upon confirming the completion of the questionnaire, their answers were automatically sent to an anonymous research server. Online data collection was implemented to minimize potential risks and allow multiple levels of confidentiality to be maintained [13]. It also meant that the possible feeling of obligation or coercion of the respondents to participate in the study was avoided and the fear of possible retaliation from superiors was alleviated [13]. Due to the COVID-19 pandemic, the entry of unemployed persons into the mentioned hospital, except for the purpose of treatment, was prohibited, thus preventing the researchers to collect data in person. Yet, online data collection increased the number of voluntary respondents owing to convenient participation in the study. The online form was sent to a total of 300 email addresses. A total of 203 completed questionnaires were received on the research server, which makes a response rate of 67.6%. All anonymous questionnaires were completely filled out and analyzed, which makes a final relevant sample of 203 respondents according to the G*Power software.

### 2.5. Ethical Consideration 

Participation in the study was voluntary and anonymous. Along with the questionnaire the respondents also received an introductory text containing information about the details of the study (objective, procedure, confidentiality, rights, and voluntariness). The respondents confirmed their voluntary participation in the study by returning the completed questionnaire to the anonymous research server. Respondents had the right to withdraw from the study without any consequences. Anonymity of the respondents was guaranteed, i.e., it was not possible to determine their identity from their answers. Only the researchers had access to the study data. The study was conducted in accordance with the approval of the Ethics Committee (No: R1-4960-4/2022).

### 2.6. Data Analysis

Categorical data were represented by absolute and relative frequencies. The normality of distribution of numerical variables was tested using the Shapiro–Wilk test. Numerical data were described by the median value and the limitations of the interquartile range. The correlation of numerical variables was measured using Spearman correlation coefficient ρ (rho). Cronbach Alpha coefficient was used to verify the internal reliability of each questionnaire. Regression analysis (bivariate and multivariate) examined which factors cause higher stress levels and poor emotional control [1,2]. All *p* values were two-sided. Significance level was set at Alpha = 0.05. Computer software used for statistical analysis were MedCalc^®^ Statistical Software version 20.100 (MedCalc Software Ltd., Ostend, Belgium; https://www.medcalc.org; accessed on 22 July 2022), and SPSS Statistics for Windows, Version 23.0 (Released 2015. IBM. IBM Corp.: Armonk, NY, USA).

## 3. Results

### 3.1. Sociodemographic Characteristics of Respondents

203 respondents, of whom 40 (19.7%) men and 162 (80.3%) women, were included in the research. There were 137 (67.5%) nurses and 66 (32.5%) physicians. The average age of respondents was 42 years (interquartile range 35 to 50) with a range of 21–72 years. In total, 76 (37.4%) respondents had a working experience of 11–20 years. In total, 174 (85.7%) respondents work in a team, and 54 (26.6%) usually in rotating shifts. (Table 2). 

### 3.2. Experienced Stress and the Ability of Emotional Regulation and Control

The overall mean of stress level experienced by the respondents according to the Questionnaire on Work-related Stressors in Healthcare Professionals (range answer 0–100) was 52.7 (interquartile range 42.6 to 62.2) with a range of 12.8–92.6 (Table 2). The highest prevalence of experienced stress (values > 60) was noticed in the Workplace Organization and Financial Issues domain in as many as 130 (64.0%) respondents, and in the Public Criticism domain in 107 (52.7%) respondents (Table 3).

According to the questionnaire, the overall mean of respondents’ emotional regulation and control ability (answer range 20–100) was 55 (interquartile range 48 to 61) (Table 4).

### 3.3. Correlation between Experienced Stress and the Ability of Emotional Regulation and Control

The experienced stress scale and the ERC scale were significantly related in all domains and in total scale scores, except for the Shift work domain, which is not significantly related to the Memory of Emotionally Saturated Content domain and the Effect of Emotions and Moods domain (Table 5). All connections are positive and weak (Rho < 0.5). The total scale of experienced stress was higher if the scale of emotional regulation and control was rated higher, i.e., the weaker regulation and control, the more severely experienced stress (Rho = 0.308; *p* ≤ 0.001) (Table 5).

### 3.4. Contribution of Specific Factors to Severe Stress Experience 

Probability to experience severe stress as a dependent variable and its relation to various predictors was analyzed using logistic regression (all respondents with a total score of experienced stress greater than 60 on a scale of 0–100). Predictor variables included general characteristics of respondents and the ERC scale domains. Multivariate logistic regression was used to structure the prediction model of severely experienced stress. *Stepwise* logistic regression was used to select predictors. The criterion of statistical significance greater than 0.10 was applied. The criterion used in this study had a threshold of 0.20. Modeling showed that the Memory of Emotionally Saturated Content domain was the only predictor. If this domain is prevalent, the respondents have a higher chance/risk (odds ratio 1.18; 95% CI 1.07 to 1.30) to experience severe stress. The model is statistically significant and explains 11.2% (according to Negelkerke) of the variance of severe stress. Additionally, it correctly classified 72.4% of cases (Table 6).

Linear logistic regression, applied to examine the contribution of a particular domain of emotional regulation and control to overall experienced stress, used overall experienced stress as the dependent variable and domains of the ERC scale as predictor variables. The results of regression analysis indicated the importance of the Memory of Emotionally Saturated Content domain as a predictor of severe stress. As a model it explained 8.8% of the change in overall experienced stress. 

The Memory of Emotionally Saturated Content domain was the significant predictor of stress levels in the Workplace Organization and Financial Issues domain (explained 5.0% of changes in stress perception), the Public Criticism domain (explained 7.3% of changes in stress perception), and the Conflicts and Communication at Work domain (explained 10.7% of changes in stress perception). Effect of Emotions and Moods was the significant predictor of the Dangers and Harms domain (explained 3.3% of changes in stress perception) and the Professional and Intellectual Demands domain (explained 8.4% of changes in stress perception) (Table 7). Memory of Emotionally Saturated Content explained 5% Workplace Organization and Financial Issues, 6.9% Public Criticism, 10.7% Conflicts and Communication at Work, and 8.8% Stress of the scale total (Table 7).

## 4. Discussion

The purpose of this study was to gain insight into the prevalence and level of experienced stress and the ability of emotional regulation and control in health professionals at the mentioned healthcare institution. Additionally, this study tried to examine the contribution of the ability of emotional regulation and control in explaining stress experienced by healthcare professionals in the hospital. 

### 4.1. Experienced Stress and Ability of Emotional Regulation and Control

The results of this study show that the overall mean of experienced stress level was 52.7 (range 0–100). This result may seem not overly worrying. Additionally, similar results of the overall stress level were described by a research team from China who examined the stress in healthcare professionals in several COVID hospitals [14]. The researchers attributed the results to increased altruism and professional commitment of healthcare professionals in moments of acute care. However, a deeper analysis of the results revealed a high percentage of healthcare professionals who were exposed to highly intense specific stressors [14]. Although deeper analysis of this study’s results also showed the significant impact of specific stressors, especially among respondents working in intensive care units (ICU, ER), it was not an unexpected result. Other studies describe a significantly more stressful impact of the hospital work environment on the occurrence of stress, especially among healthcare professionals who care for terminally ill patients [15,16,17,18]. In this study, the highest prevalence of 64% of experienced stress was found in the Organization and Financial Issues domain, followed by 52.7% in the Public Criticism domain, which was also not a surprising result. Unforeseen situations, associated with poor workplace organization, are very stressful for respondents and indirectly imply work reorganization as the main stress prevention strategy [19]. High level of stress due to staff shortage was expressed by as many as 45.3% of respondents. In total, 38.9% of respondents experienced stress due to work restrictions caused by financial issues and the impossibility of applying the most appropriate therapy. This is also supported by the results of other studies [20,21]. 72.9% of respondents experienced stress in the Public Criticism domain, which is in accordance with other studies [22,23]. The fact that the relationship between healthcare professionals and patients has become more complex was confirmed by 78.8% of respondents who experienced exposure to inappropriate public criticism as stressful; 69% of respondents consider the responsibility during 24 h shifts to be significantly stressful. Therefore, taking all study results into consideration, it is necessary to establish a dynamic and high-quality professional-patient relationship that will significantly contribute to the satisfaction of both parties [24]. Furthermore, it will simultaneously lead to economic improvements for patients and the entire healthcare system [22].

The overall mean of the ability of emotional regulation and control in this study was 55 (response range 20–100), which indicates that respondents generally assess their ability of emotional control and regulation at workplace as insufficient. Similar results were described by another study [25], which may indicate poor emotional regulation in helping occupations, possibly due to their emotional overload. A more detailed results analysis indicated that 42.4% of respondents feel hopeless in unpleasant situations. Primary emotion to a negative event can be explained with the clarity of feelings, i.e., understanding feelings prevents further escalation of uncontrolled emotional reactions [8]. Furthermore, when in a bad mood 35.5% of respondents mostly noticed only bad things. Yet, 47.3% of them had no problems carrying through with the work in such a mood. Ways of overcoming problems in crisis situations are the result of the level of awareness of personal feelings as a measure of emotional achievement. Moreover, they also serve as the basis for developing emotional intelligence. The obtained results showed that slightly less than half of the respondents come to work visibly in a bad mood and probably under stress from their private lives; nevertheless, they perform their tasks without major problems. It is evident that the respondents strive to provide patient-centered high-quality healthcare while successfully regulating their emotions. This represents the most complex level of emotional intelligence which leads to further emotional and intellectual advancement of an individual [8]. The literature describes the concepts of meta-evaluation and meta-regulation, which should also be considered when interpreting the results of this study [26]. These concepts include the attention an individual pay to the development of clarity of feelings and acceptability of mood and their influence on the way of thinking [27]. In the case of anger, 36% of respondents stated that “they do not see red”, that is, 42.4% of them notice all the events around them properly when they are angry. According to Takšić et al. [27], an individual experience feeling that are interconnected and depend on circumstances. Anger expression may result in personal satisfaction or feeling of guilt, depending on the situation. Therefore, less than half of the respondents control their emotions in anger and perform tasks without problems. However, the tasks are often performed routinely, which is supported by the fact that only 16.8% of respondents have a strong influence on thinking. Moreover, 71.8% of respondents stated that a bad mood does not affect their regular and dedicated task performance. An important element of emotional intelligence is the concept of understanding the course of the development of emotions in interpersonal relationships [8,27]. This study found that 67.4% of respondents understand the course of the development of emotions, as they never yell at a person who has done something wrong when in a state of anger. The literature describes the concept of anger as a mobilizing force that can change the state of emotional blockage and, eventually, result in satisfaction [28]. 

41.9% of respondents only sometimes immediately react violently in a state of anger and rage, while 54.4% of them declared that they always have their feelings under control. Recent theories confirm that thinking processes are led by feelings which consequently motivate adaptive behavioral activities [29]. In moments of anger or sadness, 42% of respondents easily forgive those who caused those feelings, which shows the initiation of cognitive activity, i.e., openness to their own and other people’s emotions, which demonstrates a high level of emotional intelligence. Furthermore, 67.8% of respondents estimated that they mostly do not remember events that are associated with negative emotions. This fact can be explained by the so-called adaptive model, in which healthcare professionals develop stress-coping mechanisms to withstand highly stressful work overload.

### 4.2. Correlation between Experienced Stress and Ability of Emotional Regulation and Control

The results of the analysis of the correlation between workplace stressors and respondents’ ability to regulate and control their emotions revealed specific relationships. Stressors from the domains of the applied Questionnaire on Workplace Stressors are statistically significantly related to all domains of the ERC scale. The domain of Shift Work is the only one not significantly related to the Memory of Emotionally Saturated Content domain and the Effect of Emotions and Moods domain, which is also supported by other researchers in their studies [1,3]. Additionally, the researchers singled out the most frequent and intense organizational and financial stressors in medical staff, e.g., unplanned 24 h shift, staff shortage, lack of time to complete tasks, work during breaks, too much administrative work, overtime, and shift work [1,3]. In addition, previous studies showed that workplace stress can have an adverse effect on the work efficiency of healthcare professionals, which directly impairs the quality of healthcare and the safety of patients [1,30].

Furthermore, the results of this study show that the Experienced Stress scale is significantly correlated with the Emotional Regulation and Control scale in all their domains and in overall scores. This indicates the correlation between more severely experienced stress with weaker emotional regulation. Accordingly, certain factors contribute more significantly to the level of experienced stress. If respondents have a stronger memory of emotionally saturated content, they also have a greater chance of experiencing severe stress. In their daily work with patients in the hospital environment, healthcare professionals are exposed to emotionally saturated content, such as compassion, inadequate empathy towards chronic patients, death, patient’s family members. Situations related to communication with patients and other healthcare professionals also belong to emotionally saturated content.

According to Pennebaker [5], the inhibition of emotions is potentially unhealthy because it serves as a cumulative stress stimulus and prevents cognitive-affective assimilation processes. Not assimilated stressful events remain in the person’s consciousness as unwanted and repetitive (ruminating) thoughts. According to the Zeigarnik effect, people tend to remember unfinished tasks [5], hence unassimilated stressful events are more likely to remain in consciousness and provoke such thoughts. Studies also confirm that ruminating about stressful events leads to negative emotions and cognitive and behavioral avoidance of the cause of stressful episodes [5]. The results of this study also indicate that the assimilation of stressful events is a significant predictor of increased stress experience in the Work Organization and Financial Issues domain, Public Criticism domain, and Conflicts and Communication at Work domain. The Work Organization and Financial Issues domain is described through 11 items, where severe stress prediction can be manifested through each of them. Some researchers suggest that the greater the number of daily problems, the greater the number of psychological symptoms experienced [31]. Several authors describe the impact of low-intensity everyday stressors as stronger than the impact of major life events that are generally assessed as highly stressful [31]. It is extremely important to note that health professionals make more mistakes under stress, and their mistakes are often fatal. Therefore, it is important to minimize the stressors to which healthcare professionals are exposed. In this study, the prediction of strongly experienced stress is also present in the Public Criticism domain, which includes the possibility of a lawsuit, conflict with patients or their family members, inadequate expectations from the patient, mistakes in informing the patient, and professional and private life. Knežević [32] stated that public criticism and lawsuits rank high as stressors, which indicates that there is insufficient patient-healthcare professional interaction, as well as inadequate communication between the media and healthcare. 

Regression analysis results of the Conflicts and Communication at Work domain indicate a possible prediction of experienced stress during communication with superiors, communication with colleagues, conflicts with superiors, colleagues, and other co-professionals. The literature [25] describes that nurses with a high level of self-control are more open in their communication with patients, who then become more inclined to share their concerns and feelings with them. Nurses understand the patient better, discuss their concerns and help them by showing respect. Such nurses develop a positive connection and help patients to maintain emotional interaction [33]. Conscious and emotionally controlled communication is the result of well-developed emotional intelligence.

### 4.3. Contribution of Specific Factors to Strongly Experienced Stress 

Emotional exhaustion is a fundamental dimension of burnout syndrome [1,30]. The effect of emotions in healthcare professionals is defined by the ability to empathize and be objective about in-hospital relationships, which affects mood and the experience of stress [34]. Weilenmann et al. [35] explained that emotional regulation may foster empathy-related emotions that can lead to improved compassion satisfaction for healthcare professionals who regularly experience emotionally challenging work. The results of this study indicate that the effect of emotions and mood is a significant stress predictor in the Dangers and Harms at Workplace domain and Professional and the Intellectual Demands domain. Danger and harms at work include radiation, inhaled anesthetics, cytostatics, infection, sharp objects, and terminal patient care. Knežević [32] indicated that nurses have a more pronounced fear of infection, cytostatics, ionizing radiation, and inhaled anesthetics than physicians who are more educated about the dangers to which they are exposed and about methods of protection at work. However, this fear could be strongly related to the effect of emotional stress that nurses experience working directly with an infected patient or administering cytostatic therapy, and less to ignorance, which can become the basis for future studies. Re-experiencing an emotionally stressful experience leads to exhaustion and dissatisfaction at workplace. Tuna and Baykal [36] suggested that the reasons for oncology nurses’ dissatisfaction with their workplace include terminally ill patients, high mortality rate, negative physical conditions in the work environment, shortage of work, and staff shortage. Some researchers found that oncology nurses face problems due to an increasing number of patients, insufficient protective measures during the preparation of antineoplastic drugs, and an overall increase in workload [37]. Consequently, it has been emphasized that all these negative effects increase the level of work-related stress in oncology nurses, and subsequently the level of burnout [38]. Furthermore, according to the results of this study, the Effect of Emotions and Moods is also a predictor of experiencing more severe stress. New technologies, new information, lack of education, and unavailability of scientific literature cause stress since emotional exhaustion and negative moods have a negative impact on professional development of healthcare professionals. According to Maslow’s hierarchy of needs, respect and self-actualization are at the very top, and if there is a negative emotional effect present in daily work, an individual will not have emotional strength to achieve new professional goals, be creative at work, accept new facts, or find problem solutions. Digitization and the introduction of new information technologies led to new term, technostress, which defines the impact of technology on the experience of increased stress among healthcare professionals. Golz et al. [39] showed that technostress has a relevant correlation with long-term consequences for staff, especially for those with burnout symptoms. Additional digital competence will be needed as a technostress inhibitor, which will enable healthcare professionals to sustainably deal with it and thus reduce the risk of long-term consequences [39].

Finally, it is necessary to look at the possibilities of emotional regulation and control training in order to improve the prediction and prevention of stressful experiences. The approach applied in this study is an important and indispensable component of the development of institutional support mechanisms for professionals. Kharatzadeh et al. [25] assessed the effectiveness of emotional regulation training on cognitive-emotional regulation strategies, depression, anxiety, stress, and the professional quality of life for intensive and critical care nurses. Their results showed that emotional regulation training can increase compassion satisfaction in nurses. The study also provided evidence that emotional regulation training may reduce burnout for intensive and critical care healthcare professionals [25], similar to the Jackson-Koku and Grime findings, which supported the association between emotional regulation and burnout in physicians [40].

### 4.4. Limitations and Recommendations for Further Research

In this cross-sectional study, the respondents were healthcare professionals who work in direct patient care and are employed at the Clinical Hospital Centre Osijek, in Osijek, Croatia. Therefore, the sample is not large enough to generalize the results of all healthcare professionals in hospital institutions throughout the Republic of Croatia. In the future, a study should be planned with more respondents from different hospital institutions (general medicine clinics and clinical centers) and from more regions in Croatia. Additionally, the majority of respondents were females with higher sensitivity to emotions/stress, which may have contributed to study findings. In addition, this study indicated the limit value of the reliability of the third domain of the ERIK scale, which is also described by the authors of the original version of the scale. However, overall questionnaire reliability in this study indicated high reliability. Furthermore, future studies could include the impact of each questionnaire item from the statistically significant domains on emotional control and regulation in healthcare professionals. This would contribute to a higher level of objectivity and a better understanding of specific stressors in the hospital environment.

### 4.5. Usefulness and Applicability of Study Results

The results of this study indicate the possibility of future predictions and prevention of stressful events in healthcare professionals. Therefore, designed prediction models significantly help healthcare institutions to create, design, and review emotional regulation strategies and training, as well as individualized programs of institutional support. The prediction model can be used by hospital management, but also by experts for the purpose of constant observation and assessment of the conditions, and possible changes in healthcare professionals. In addition, based on the results of this study, an employment testing and screening program can be designed and implemented. The results of such tests would provide insight into the specific individual characteristics, wishes, and needs of candidates, which would significantly contribute to more efficient allocation of individual jobs. This individualized approach significantly contributes to work satisfaction, and minimizes work-related stress of healthcare professionals, which ultimately leads to improved quality of healthcare. 

## 5. Conclusions

The results of this study indicate a high percentage of exposure of healthcare professionals to very intense specific stressors, especially in the Work Organization and Financial Issues domain, as well as in the Public Criticism domain. Respondents generally assess their ability to control and regulate emotions in the workplace as insufficient. Low ability of emotional regulation and control also predicts higher prevalence of experienced stress in healthcare professionals. Memory of Emotionally Saturated Content and Effect of Emotions and Moods significantly contribute to a higher occurrence of stressful factors. Re-experiencing emotional stress leads to dissatisfaction and exhaustion in healthcare professionals, which ultimately negatively affects patient satisfaction and the quality of healthcare. The results of this study indicate the need to establish effective and organized institutional support aimed at objectifying stress and strengthening emotional intelligence and empathy in healthcare professionals. Due to long-term neglect of these issues, hospital systems are increasingly faced with the outflow of best physicians and nurses to private health institutions or situations where nurses change their careers.

## Figures and Tables

**Table 1:** Description of domains and items of the Questionnaire on Workplace Stressors for Hospital Professionals.

Domains	Number of Items	Description
1. Workplace Organization and Financial Issues	11	Small number of employees, inadequate income, unforeseen situations, financial restrictions, work overload, administrative work, time limit, inadequate workspace, impossibility of promotion, deadlines, poor organization.
2. Public Criticism	6	Lawsuit, public criticism, conflicts with patients or their family members, inadequate expectations from patients, misinformed patients, professional and private life.
3. Dangers and Harms at Workplace	5	Radiation, inhaled anesthetics, cytostatics, infection, sharp objects, terminally ill patients
4. Conflicts and Communication at Work	5	Communication with superiors, communication with colleagues, conflicts with superiors, conflicts with colleagues, conflicts with other co-workers
5. Shift Work	5	Overtime, shift work, night work, 24 h shift
6. Professional and Intellectual Demands	4	New technologies, new information, lack of education, unavailability of literature

**Table 2:** Sociodemographic Characteristics of Respondents (*n* = 203).

Characteristics of Respondents	Number (%)
Sex	
Male	40 (19.7)
Female	163 (80.3)
Workplace	
ICU, Emergency Room (ER), Psychiatry, Oncology	39 (19.2)
Other departments	164 (80.8)
Work experience (years)	
0–5	23 (11.3)
6–10	21 (10.3)
11–20	76 (37.4)
21–30	43 (21.2)
>30	40 (19.8)
Education	
Nurses	137 (67.5)
Physicians	66 (32.5)
Marital status	
single	43 (21.2)
married	126 (62.1)
partnered	19 (9.4)
divorced	10 (4.9)
widowed	5 (2.5)
Work shift	
morning shift	42 (20.7)
afternoon shift	1 (0.5)
two shifts (morning, afternoon)	19 (9.4)
three shifts (morning, afternoon, night)	36 (17.7)
morning shift and 24 h shift	51 (25.1)
other (rotating shift)	54 (26.6)
Work	
team	174 (85.7)
individual	29 (14.3)
Total	203 (100)

**Table 3:** Respondents according to stress level (N = 203).

Domain	Level of Stress	
	No Stress (≤60)	Stressful (>60)
	*n* (%)	*n* (%)
Workplace Organization and Financial Issues	73 (36.0)	130 (64.0)
Public Criticism	96 (47.3)	107 (52.7)
Dangers and Harms at Workplace	172 (84.7)	1 (15.3)
Conflicts and Communication at Work	146 (71.9)	57 (28.1)
Shift Work	152 (74.9)	51 (25.1)
Professional and Intellectual Demands	166 (81.8)	37 (18.2)

**Table 4:** Level of emotional regulation and control ability of respondents (N = 203).

Domain	Ability of Emotional Regulation		
	Number of Statements	Range of Points *	Median (Interquartile Range)
Effect of Emotions and Moods	8	8–40	21 (18–25)
Memory of Emotionally Saturated Content	6	6–30	18 (15–20)
Control of Personal Emotional Reactions	6	6–30	15 (13–18)
Total ERIK scale	20	20–100	55 (48–61)

* higher point value = lower emotional regulation and control ability.

**Table 5:** Correlation between experienced stress and the ability of emotional regulation and control.

Stress Domains	ERC Scale Domains			Total ERC Scale
	Effect of Emotions and Moods	Memory of Emotionally Saturated Content	Control of Personal Emotional Reactions	
	Spearman’s Correlation Coefficient			
Organization and Financial Issues	0.167 *	0.237 ***	0.191 *	0.211 ***
Public Criticism	0.165 *	0.283 ***	0.196 *	0.235 ***
Dangers and Harms	0.193 *	0.205 ***	0.149 *	0.222 ***
Conflicts and Communication at Work	0.213 ***	0.345 ***	0.272 ***	0.304 ***
Shift work	0.135	0.135	0.156 *	0.146 *
Professional and Intellectual Demands	0.285 ***	0.272 ***	0.187 **	0.292 ***
Total stress scale	0.257 ***	0.317 ***	0.260 ***	0.308 ***

* *p* value < 0.05; ** *p* value < 0.01; *** *p* value < 0.001.

**Table 6:** Predicting probability of severe stress (bivariate and multivariate regression analysis).

Bivariate Regression				
General Characteristics of Respondents	ß	Wald	*p* Value	OR (95 % CI)
Sex (F)	0.06	0.02	0.87	1.06 (0.50–2.26)
Age	−0.01	0.20	0.66	0.99 (0.97–1.02)
Marital status (unmarried)	−0.18	0.35	0.55	0.83 (0.45–1.54)
Number of children	0.06	0.08	0.77	1.06 (0.72–1.56)
Level of education (physicians *)				
Nurses	0.07	0.05	0.83	1.08 (0.56–2.06)
Total work experience	−0.002	0.02	0.88	0.99 (0.97–1.02
Work experience at present position	0.003	0.04	0.85	1.01 (0.97–1.03)
Employment status (fixed-term *)	1.05	1.82	0.18	2.86 (0.62–13.2)
Emotional regulation and control scale				
Effect of Emotions and Moods	0.10	10.99	<0.001	1.11 (1.05–1.17)
Memory of Emotionally Saturated Content	0.15	13.17	<0.001	1.66 (1.07–1.27)
Control of Personal Emotional Reactions	0.14	9.85	0.002	1.15 (1.06–1.26)
Total of emotional regulation and control scale	0.06	14.63	<0.001	1.06 (1.03–1.09)
Multivariate regression (Stepwise method)				
Memory of emotionally saturated content	0.16	10.46	0.001	1.18 (1.07–1.30)
Constant	−3.72	15.3	<0.001	

ß—regression coefficient; * reference value/group.

**Table 7:** Functional correlation between overall experienced stress and emotional regulation and control (multivariate linear regression, Stepwise method).

	ß	*p*	95% CI (ß)	Model Summary
Workplace Organization and Financial Issues				R = 0.233
Memory of Emotionally Saturated Content	0.94	<0.001	0.39–1.48	R^2^ = 0.055
Constant	48.94	0.001	39.1–58.8	R^2^ correction = 0.050
Public Criticism				R = 0.271
Memory of Emotionally Saturated Content	1.40	<0.001	0.71–2.09	R^2^ = 0.073
Constant	36.64	<0.001	24.04–49.2	R^2^ correction = 0.069
Dangers and Harms				R = 0.195
Effect of Emotions and Moods	0.71	0.005	0.21–1.20	R^2^ = 0.038
Constant	21.94	<0.001	10.9–32.9	R^2^ correction = 0.033
Conflicts and Communication at Work				R = 0.334
Memory of Emotionally Saturated Content	1.97	<0.001	1.19–2.74	R^2^ = 0.112
Constant	21.94	0.08	−1.29–26.8	R^2^ correction = 0.107
Shift Work				
No significant model	-	-	-	
Professional and Intellectual Demands				R = 0.298
Effect of Emotions and Moods	0.83	<0.001	0.46–1.21	R^2^ = 0.089
Constant	27.92	<0.001	19.7–36.2	R^2^ correction = 0.084
Stress scale total				R = 0.304
Memory of Emotionally Saturated Content	1.11	<0.001	0.63–1.59	R^2^ = 0.092
Constant	32.74	<0.001	23.92–41.6	R^2^ correction = 0.088

ß—regression coefficient, R—Pearson correlation coefficient.

## Data Availability

All data generated analyzed during the current study are available from the corresponding author on reasonable request.
